# Relapsing low-flow alarms due to suboptimal alignment of the left ventricular assist device inflow cannula

**DOI:** 10.1093/ejcts/ezac415

**Published:** 2022-08-22

**Authors:** Casper F Zijderhand, Wiebe G Knol, Ricardo P J Budde, Cornelis W van der Heiden, Kevin M Veen, Jelena Sjatskig, Olivier C Manintveld, Alina A Constantinescu, Ozcan Birim, Jos A Bekkers, Ad J J C Bogers, Kadir Caliskan

**Affiliations:** Department of Cardiothoracic Surgery, Thoraxcenter, Erasmus MC, University Medical Center Rotterdam, Rotterdam, Netherlands; Department of Cardiology, Thoraxcenter, Erasmus MC, University Medical Center Rotterdam, Rotterdam, Netherlands; Department of Cardiothoracic Surgery, Thoraxcenter, Erasmus MC, University Medical Center Rotterdam, Rotterdam, Netherlands; Department of Radiology and Nuclear Medicine, Erasmus MC, University Medical Center Rotterdam, Rotterdam, Netherlands; Department of Radiology and Nuclear Medicine, Erasmus MC, University Medical Center Rotterdam, Rotterdam, Netherlands; Department of Cardiology, Thoraxcenter, Erasmus MC, University Medical Center Rotterdam, Rotterdam, Netherlands; Department of Cardiothoracic Surgery, Thoraxcenter, Erasmus MC, University Medical Center Rotterdam, Rotterdam, Netherlands; Department of Cardiothoracic Surgery, Thoraxcenter, Erasmus MC, University Medical Center Rotterdam, Rotterdam, Netherlands; Department of Cardiology, Thoraxcenter, Erasmus MC, University Medical Center Rotterdam, Rotterdam, Netherlands; Department of Cardiology, Thoraxcenter, Erasmus MC, University Medical Center Rotterdam, Rotterdam, Netherlands; Department of Cardiothoracic Surgery, Thoraxcenter, Erasmus MC, University Medical Center Rotterdam, Rotterdam, Netherlands; Department of Cardiothoracic Surgery, Thoraxcenter, Erasmus MC, University Medical Center Rotterdam, Rotterdam, Netherlands; Department of Cardiothoracic Surgery, Thoraxcenter, Erasmus MC, University Medical Center Rotterdam, Rotterdam, Netherlands; Department of Cardiology, Thoraxcenter, Erasmus MC, University Medical Center Rotterdam, Rotterdam, Netherlands

**Keywords:** Heart failure, Left ventricular assist device, Low-flow alarms, Inflow cannula, Contrast-enhanced computed tomography

## Abstract

**OBJECTIVES:**

This retrospective study investigated the correlation between the angular position of the left ventricular assist device (LVAD) inflow cannula and relapsing low-flow alarms.

**METHODS:**

Medical charts were reviewed of all patients with HeartMate 3 LVAD support for relapsing low-flow alarms. A standardized protocol was created to measure the angular position with a contrast-enhanced computed tomography scan. Statistics were done using a gamma frailty model with a constant rate function.

**RESULTS:**

For this analysis, 48 LVAD-supported patients were included. The majority of the patients were male (79%) with a median age of 57 years and a median follow-up of 30 months (interquartile range: 19–41). Low-flow alarm(s) were experienced in 30 (63%) patients. Angulation towards the septal–lateral plane showed a significant increase in low-flow alarms over time with a constant rate function of 0.031 increase in low-flow alarms per month of follow-up per increasing degree of angulation (*P* = 0.048). When dividing this group using an optimal cut-off point, a significant increase in low-flow alarms was observed when the septal–lateral angulation was 28° or more (*P* = 0.001). Anterior–posterior and maximal inflow cannula angulation did not show a significant difference.

**CONCLUSIONS:**

This study showed an increasing number of low-flow alarms when the degrees of LVAD inflow cannula expand towards the septal–lateral plane. This emphasizes the importance of the LVAD inflow cannula angular position to prevent relapsing low-flow alarms with the risk of diminished quality of life and morbidity.

**Figure:**
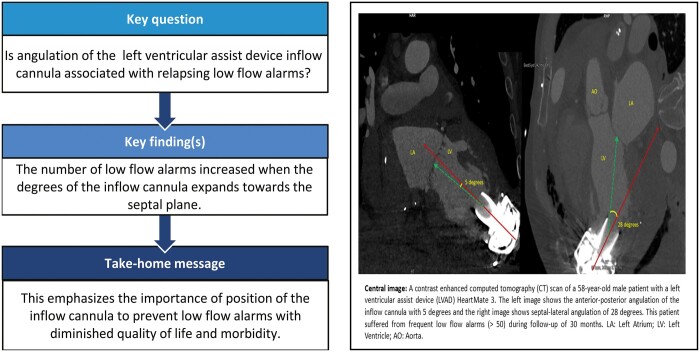

## INTRODUCTION

Left ventricular assist devices (LVAD) have become a viable option in the treatment of end-stage heart failure (HF) as a bridge to transplantation, bridge to candidacy and destination therapy [1]. LVAD treatment improves the overall survival and quality of life in patients with advanced HF [2, 3]. However, LVAD treatment introduces post-implantation complications such as bleeding, infection, thrombosis, haemodynamic alternations and device malfunction [4].

At the centre of this, the LVAD creates a continuous flow from the inflow cannula in the apex of the left ventricle through the outflow cannula into the ascending aorta. If this blood flow decreases below a pre-specified flow threshold of 2.5 l/min, the pump creates a low-flow alarm. There may be several reasons for a low-flow alarm, the most frequent is hypovolaemia, and other risk factors include hypertension, inflow or outflow graft obstruction, right HF, and arrhythmias [5]. However, some patients suffer from relapsing low-flow alarms, meaning frequent and recurrent low-flow alarms, without the clinical signs of the reasons mentioned earlier.

Since suction from the inflow cannula is a crucial aspect of generating flow, an unfavourable position or angulation of the inflow cannula could theoretically result in low flow. Angulation of the inflow cannula towards the septum, inferior or the free-wall of the left ventricle could thereby lead to suboptimal suction of the LVAD and result in relapsing low-flow alarms. However, research on both relapsing low-flow alarms and the influence of the angulation of the inflow cannula in the apex of the left ventricle is not earlier described in the literature. Therefore, this study aimed to evaluate the correlation between the inflow cannula angulation, the prevalence of relapsing low-flow alarms and eventual clinical sequelae in LVAD-supported patients.

## METHODS

### Study design and data collection

We retrospectively reviewed the hospital records for all patients who underwent a HeartMate 3 implantation in the Erasmus Medical Center between January 2016 and December 2020. All LVAD implantations at our centre are performed with a median sternotomy and extracorporeal circulation. Patients were eligible if they underwent a contrast-enhanced computed tomography (CT) scan of the chest and aged ≥18 years. In our centre, patients receive, per protocol, a (contrast-enhanced) CT scan between 3 and 6 weeks after LVAD implantation. Patient characteristics before LVAD implantation and procedural characteristics were collected from the local data of the European Registry for Patients with Mechanical Circulatory Support. Data of low-flow alarms were retrieved from the LVAD technicians data, obtained during follow-up or unexpected hospital visits. The default threshold for a low-flow alarm is set at 2.5 l/min. In a few patients, the low-flow threshold was set on 2.0 l/min due to frequent relapsing low-flow alarms with had a substantial impact on the quality of life.

### Ethics statement

Approval was obtained from the institutional medical ethical committee to conduct this study (MEC-2017-1013).

### Computed tomography analysis

**Figure 1:**
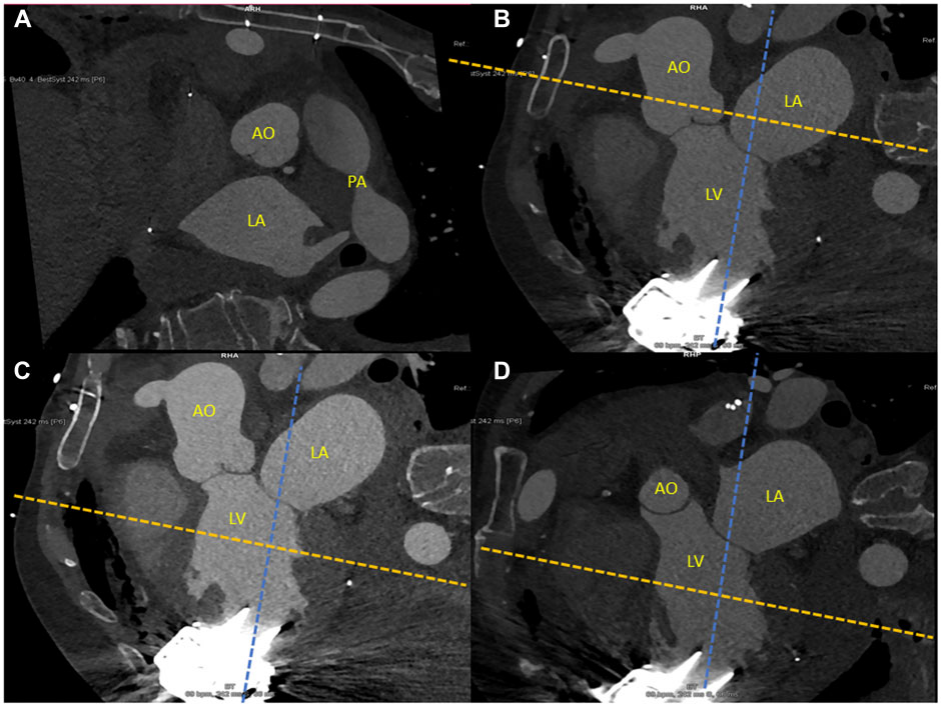
(**A**) Dashed orange square as starting plane. (**B**) One axis was then aligned between the middle of the inflow cannula and the middle of the mitral valve coaptation (blue dashed line). The third plane was rotated to the maximal diameter of the aortic root (orange dashed line). (**C**) Next, the plane was moved to the maximal left ventricular diameter (dashed orange line). (**D**) The plane was re-rotated to the middle of the interventricular septum, with a similar length of the septum anteriorly and posteriorly as ‘ideal axis’ (dashed orange line). AO: aorta; LA: left atrium; LV: left ventricle; PA: pulmonary artery.

**Figure 2:**
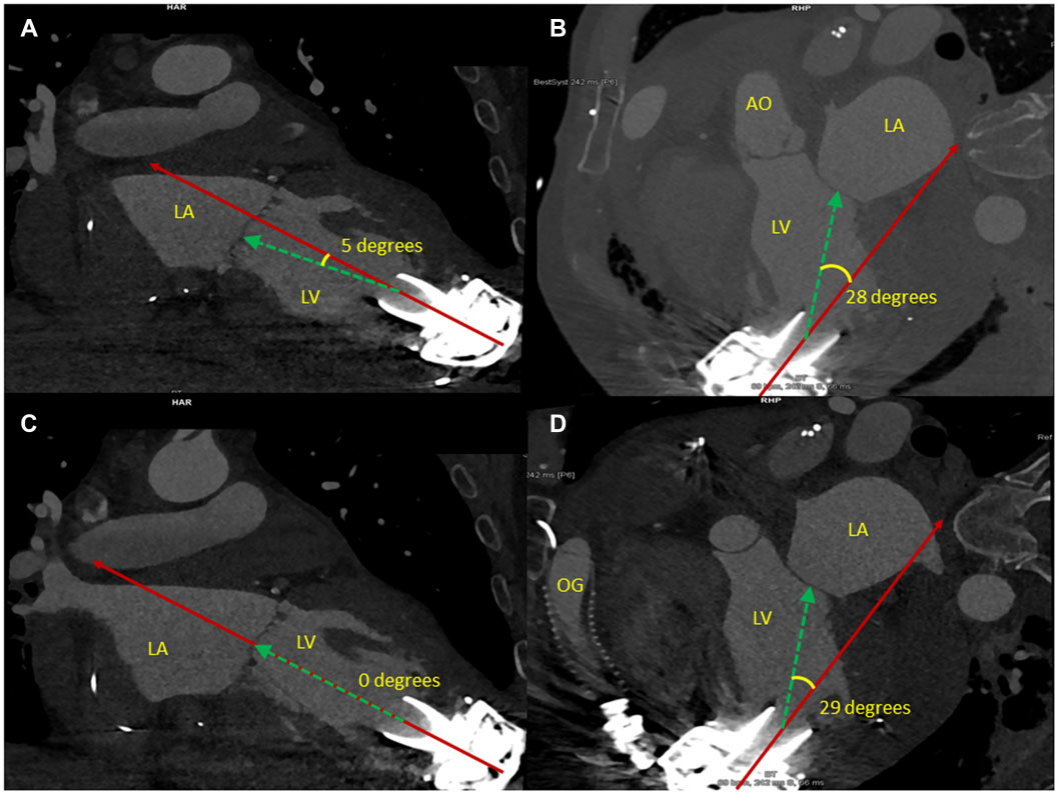
Measurement of the angulation. The green dashed line is the ‘ideal axis’ and the solid red line is the centre line of the inflow cannula. (**A**) Angulation to the septum or lateral wall. (**B**) Angulation to the anterior or posterior wall. (**C**) Finally, the plane was re-rotated once more, to a position where the angulation of the inflow cannula equalled zero. (**D**) This yielded the maximal angulation, measured in the perpendicular view. AO: aorta; LA: left atrium; LV: left ventricle; OG: outflow graft.

**Figure 3:**
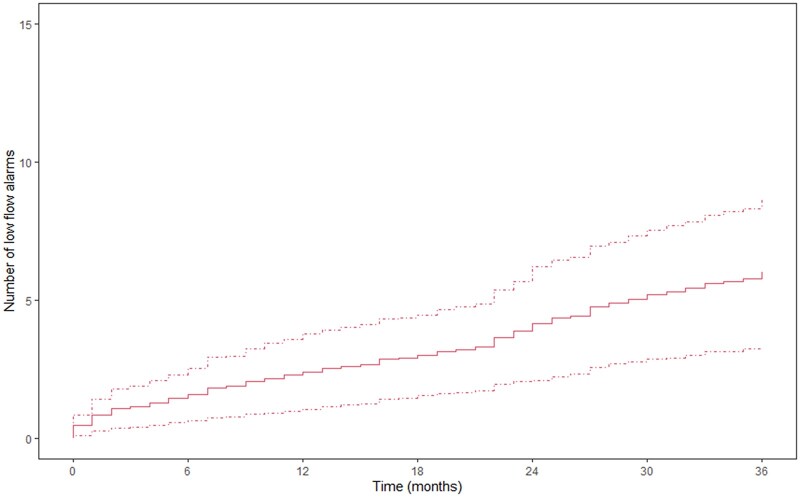
Mean cumulative function of relapsing low-flow alarms, presented with dashed lines for the confidence intervals. The *Y*-axis presents the number of low-flow alarms and the *X*-axis represents the time in months.

To prevent arbitrary measurements, a strictly standardized protocol was used to measure the inflow cannula angulation. After selecting the right scan (if multiple contrast-enhanced scans were available, biphasic scans and those earliest after surgery were prioritized), a three-chamber view of the left atrium, pulmonary artery and aorta was acquired (Fig. 1A). Biphasic scans were used to acquire images both in the systolic and diastolic phases of the heart cycle. One axis was then aligned between the middle of the inflow cannula and the middle of the mitral valve coaptation, yielding 2 dimensions parallel and perpendicular to this ‘ideal axis’. The third plane was first rotated in the plane with maximal diameter of the aortic root (Fig. 1A and B). Next, the plane perpendicular to the ‘ideal axis’ (more or less corresponding to the short axis view on ultrasound) was moved to the level of maximal left ventricular diameter (Fig. 1C). Finally, the third plane was re-rotated to the middle of the interventricular septum, leaving a similar length of septum anteriorly and posteriorly of this plane (Fig. 1D). The angulation to septum (positive) or lateral wall (negative) was then measured in 1 plane (Fig. 2A) and the angulation to anterior (positive) or posterior (negative) in the other plane (Fig. 2B). Finally, the third plane was re-rotated once more, to a position where the angulation of the inflow cannula equalled zero (Fig. 3A). This yielded the maximal angulation, measured in the perpendicular view (Fig. 3B).

### Outcomes

The primary outcome was the correlation between the inflow cannula angulation and the number of (relapsing-) low-flow alarms. Secondary outcomes were differences in angulation during the systolic and diastolic heart phases, the influence of septal–lateral or anterior–posterior inflow cannula angulation on the number of low-flow alarms and the occurrence of relapsing low-flow alarms in 2 different groups divided by the severity of angulation. Subsequently, clinical outcomes including unexpected readmission, right HF, bleeding, neurological dysfunction and mortality were compared.

### Statistical analysis

Continuous variables were presented as mean with standard deviation if Gaussian distribution and as median with interquartile range (IQR) if non-Gaussian distribution. Categorical data were presented as percentages. Normality was tested using the Shapiro–Wilk test. A comparison was made between the systolic and diastolic heart phases to evaluate possible influences on the inflow cannula angulation during the cardiac cycle, using the Kruskal–Wallis test. Differences in clinical outcomes were calculated over time and presented with hazard ratios. Low-flow alarm(s) can occur multiple times in 1 patient, affecting mean occurrence rates. To give a comprehensive overview, considering multiple recurrent events, the cumulative mean number of events over time was calculated using a non-parametric mean cumulative function and presented in a plot [6]. The variance was estimated using the Lawless and Nadeau method [7]. A gamma frailty model was created to calculate the influence of the angulation on the number of low-flow events during the follow-up period [8]. This model shows the increment or decrement in the number of low-flow alarm(s) per increasing degree of angulation of the inflow cannula. An optimal cut-off point with receiver operating characteristic (ROC) curve analysis was performed to divide the population into 2 groups according to the angulation. To understand the direction of the angulation, we calculated the optimal cut-off point for the septal–lateral, anterior–posterior angulation, and the maximal angulation in any direction separately. Again a mean cumulative incidence function was calculated and presented in a plot divided into both groups based on the optimal cut-off point depending on the plane. Statistical analyses were done in R (Version 4.1.2).

## RESULTS

### Baseline and procedural characteristics

In total, 101 patients with a HeartMate 3 LVAD were identified at our centre of which 48 met the requirements for inclusion. The median follow-up was 30 months [IQR: 19–41], with a median age of 56 [IQR: 51–62] years and 79.2% males. The most frequent aetiology of end-stage HF was dilated cardiomyopathy (52%). Patients were mainly in Interagency Registry for Mechanically Assisted Circulatory Support profiles 1–3 before implantation. Cardiac rhythm in 53.2% of the patients was sinus rhythm and 87.5% of the patients had an implantable cardioverter-defibrillator in place. The most prevalent LVAD strategy was (intended) bridge to transplantation in 60.4% of the patients and long-term destination therapy in 33.3% of the patients. The median cardiopulmonary bypass time was 97 min [IQR: 79–116], and the median time in the operating room for implant was 318 min [IQR: 290–386]. The median length of intensive care unit stay was 8 days [IQR: 5–12] and hospital stay was 32 days [IQR: 27–44]. The median time from LVAD implantation to initial CT scan was 6.8 weeks [IQR: 3–43] (Table 1).

**Table 1:** Baseline and procedural characteristics

	Overall (*n* = 48)
Demographics	
Age (years)	56.0 [50.8, 62.0]
Male	38 (79.2)
Height (cm)	178.0 [171.5, 183.5]
Weight (kg)	76.0 [69.7, 87.9]
Body mass index	22.1 [19.7, 25.0]
Primary diagnosis	
Dilated cardiomyopathy	25 (52)
Ischaemic heart disease	20 (41.7)
Others	3 (6.3)
INTERMACS patient profile	
1	13 (27.1)
2	10 (20.8)
3	14 (29.2)
≥4	11 (22.9)
Comorbidities	
Diabetes	8 (16.7)
ICD therapy	42 (87.5)
Neurological event	3 (6.4)
Preoperative status	
Intra-aortic balloon pump	16 (33.3)
Extra-corporeal membrane oxygenation	5 (10.4)
Intravenous inotropes	34 (70.8)
ECG rhythm	
Sinus	25 (53.2)
Atrial fibrillation	8 (17.0)
Paced	14 (29.8)
Procedural characteristics	
Device strategy	
(Possible) bridge to transplant	29 (60.4)
Destination therapy	16 (33.3)
Others	3 (6.3)
Cardiopulmonary bypass time (min)	97.0 [79.0, 116.0]
Time in operating room for implant (min)	318.0 [289.8, 385.8]
ICU stay (days)	8.0 [5.0, 12.0]
Hospital stay (days)	32.0 [27.0, 55.0]
Length of follow-up (months)	30.0 [18.8, 41.3]
Timing to CT scan (weeks)	6.8 [2.7, 42.9]

Continuous variables are depicted as median [IQR] and categorical variables are depicted as count (percentage).

CT: computed tomography; ECG: electrocardiogram; ICD: implantable cardioverter-defibrillator; ICU: intensive care unit; INTERMACS: Interagency Registry for Mechanically Assisted Circulatory Support; IQR: interquartile range.

### Outcomes

**Figure 4:**
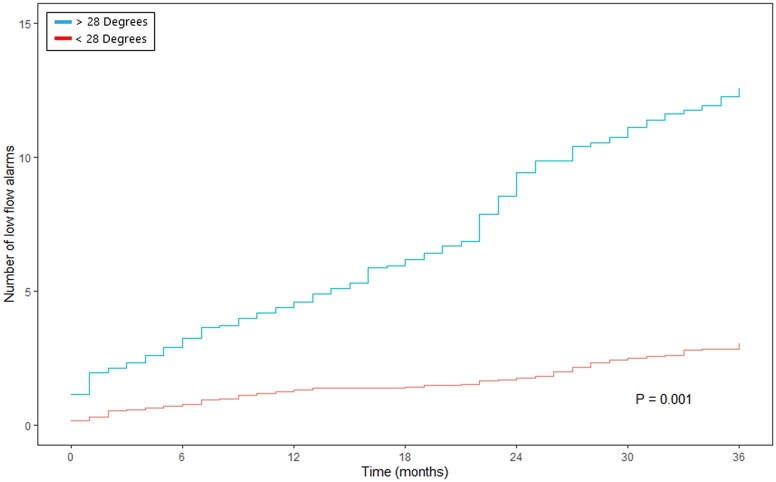
Mean cumulative function of relapsing low-flow alarms, with <28° or >28° of inflow cannula angulation in the septal–lateral direction. The *Y*-axis presents the number of low-flow alarms and the *X*-axis represents the time in months.

**Figure 5:**
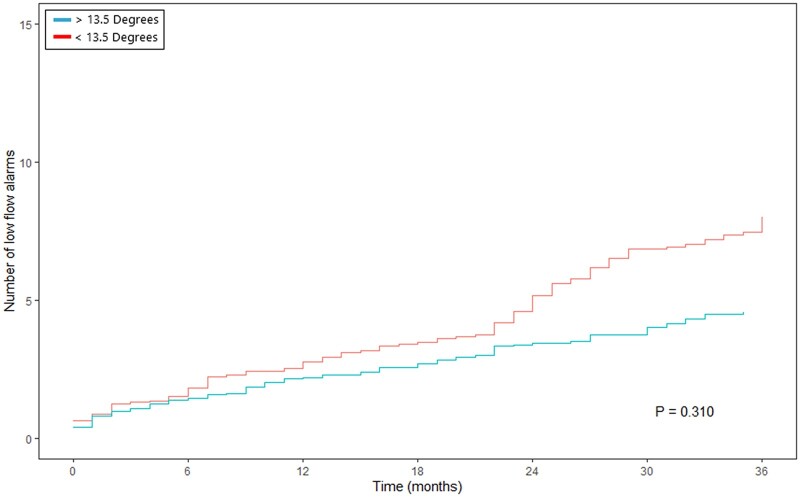
Mean cumulative function of relapsing low-flow alarms, with <13.5° or >13.5° of inflow cannula angulation in the anterior–posterior direction. The *Y*-axis presents the number of low-flow alarms and the *X*-axis represents the time in months.

**Figure 6:**
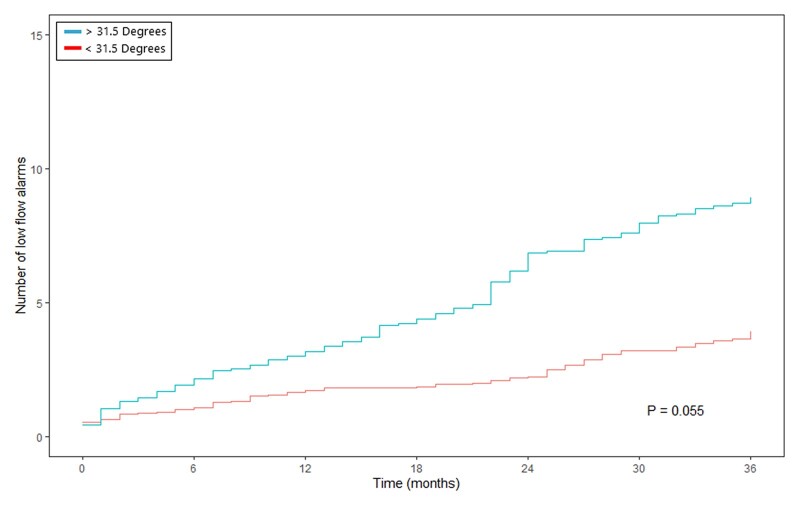
Mean cumulative function of relapsing low-flow alarms, with <31.5° or >31.5° of maximal inflow cannula angulation in any direction. The *Y*-axis presents the number of low-flow alarms and the *X*-axis represents the time in months.

Overall, 30 patients (62.5%) experienced low-flow alarm(s) during follow-up. The occurrence of low-flow alarms over time is presented in Fig. 3. This figure suggests an increment in the number of low-flow alarms during follow-up and relapsing nature. The gamma frailty model, with constant rate function for the different planar views, shows an increment of low-flow alarm(s) per month of follow-up per increasing degree of angulation in the given direction (Table 2). Septal–lateral angulation showed a significant constant rate function of 0.031 (standard error 0.016, *P*-value 0.048). Thus, for every degree of angulation, an increment of 0.031 low-flow alarm(s) per month of follow-up is expected. In addition, maximal angle in any direction and anterior–posterior angulation did not show a significant increase in low-flow alarms per degree of angulation (respectively, *P =* 0.344 and *P* = 0.967). According to the ROC analysis (Supplementary Material, Figs S1–S3), the optimal cut-off point was 28° for septal–lateral angulation, 13.5° for anterior–posterior angulation and 31.5° for the maximal angulation in any direction. Septal–lateral angulation of 28° or more showed a significant increase in the number of low-flow alarms over time (Fig. 4). Both maximal angulation in any direction and anterior–posterior angulation did not show a significant increase in the number of low-flow alarms (Figs 5 and 6). However, maximal angulation of the inflow cannula in any direction showed a trend towards significance (*P* = 0.055). When dividing the patients according to the systolic and diastolic heart phases, no differences were seen between the angulation in the different planes (Supplementary Material, Table S1). Clinical outcomes during follow-up, including unexpected readmission, right HF, bleeding, neurological dysfunction and mortality, did not differ when dividing the cohort according to the optimal cut-off points for each plane (Supplementary Material, Table S2).

**Table 2:** Gamma frailty model with constant rate function of the number of low-flow alarms per month of follow-up per 1 increasing degree of angulation

Degrees of angulation	Constant rate function (number of low-flow alarms per month of follow-up per 1 increasing degree of angulation)	SE	*P*-Value
Septal–lateral	0.031	0.016	0.048
Anterior–posterior	<0.001	0.013	0.967
Maximal inflow cannula angulation in any direction	0.015	0.016	0.344

Presented with SE and *P*-value.

SE: standard error.

## DISCUSSION

In this article, we analysed the association between the inflow cannula angulation and relapsing low-flow alarms in patients with an LVAD, using contemporary analyses with constant rate function for recurrent low-flow alarms. The main findings were that an increase in the degrees of the inflow cannula angulation leads to an increase in low-flow alarms, specifically in, septal–lateral angulation. Moreover, septal–lateral inflow cannula angulation of 28° or more was found to be an optimal cut-off point for increased low-flow alarms over time. This implies the clinical relevance of the angular position of the inflow cannula in the apex of the left ventricle to prevent relapsing (red) low-flow alarms with possible clinical consequences.

### Relapsing low-flow alarms

Causes of relapsing low-flow alarms are poorly investigated. However, known is that hypovolaemia, hypertension, inflow- or outflow graft obstruction, right ventricular failure and arrhythmias are possible causes of relapsing low-flow alarms [5]. When a patient is dehydrated and hypovolemic, the left ventricle is feasibly decompressed whereby the inflow cannula contacts the left ventricle and suction of the LVAD is insufficient [9]. When the inflow cannula is angulated towards the septal or lateral wall of the left ventricle, it is assumable that suction from the LVAD is not optimal, resulting in low-flow alarms. Our study confirms this assumption, specifically in septal–lateral angulation of 28° or more. Cardiac remodelling might increase the incidence of low-flow alarms during long-term follow-up. Serial contrast-enhanced CT scans would be required to correctly assess the influence of cardiac remodelling on low-flow alarms. Furthermore, relapsing obstreperous red low-flow alarms could have a significant impact on the quality of life, besides the frequent need for unexpected outpatient or emergency department consultation. Earlier research showed that LVAD-supported patients with fewer adverse events tend to have a higher quality of life [10]. The noise and red warning message of low-flow alarms are meant to be taken very seriously. When a patient is suffering from relapsing low-flow alarms due to the position of the inflow cannula, the real clinical sequelae seem to be less relevant. However, our study included mainly patients who survived the index hospitalization and developed relapsing low-flow alarms. Critical conditions such as obstruction of the inflow or outflow graft presents as a low-flow alarm and is life threatening. Patients with relapsing low-flow alarms should therefore, if suitable, be instructed extensively with regard to different clinical symptoms as possible reasons for low-flow alarms and their relapsing low-flow alarms due to abnormal angulation. This emphasizes the importance of evaluating the inflow cannula angulation preoperatively to have as much as possible optimal position of the LVAD inflow cannula as well as postoperative evaluation.

### Adverse events

Our study is, to our knowledge, the first study who observed an association between the angulation of the inflow cannula and relapsing low-flow alarms. However, other clinical outcomes during follow-up did not differ in regard to angulation. A study that evaluated chest radiographs showed an increase in the composite outcome, consisting of HF readmission, low-flow alarms, stroke and inflow or outflow graft occlusion when the angle of the pump is increased [11]. Another small sample size study that evaluated echocardiographic images showed an increased occurrence of stroke when the distance between the inflow cannula duct and the left ventricular septum was larger [12]. Both studies suggest a higher risk of adverse events when the angular position of the inflow cannula increases. It is plausible to suggest that low-flow alarms are a result of suboptimal flow in the LVAD and could result in thromboembolic complications. Conceivably, an explanation for the differences concerning our study could be that we included more patients and the measurements were done with a CT scan instead of a chest X-ray, providing a more feasible overview of the inflow cannula angulation. Nevertheless, research on this subject is scarce, attention should be paid to maintaining anticoagulation in the therapeutic range to prevent thromboembolic complications in patients suffering from relapsing low-flow alarms.

### Position of the inflow cannula

Differences in position and depth of the LVAD inflow cannula are an actual topic of research [11–14]. The position of the inflow cannula is a crucial part of LVAD implantation to prevent long-term, adverse events such as thromboembolic events [5, 14]. During LVAD implantation, our surgeons identified the left ventricular apex based on visual and palpable landmarks for the maximal inflow cannula position. Subsequently, transoesophageal guidance is used to optimize the placement of the inflow cannula [15]. Thereby, other surgical techniques, including left thoracotomy, could give surgeons a better vision and alignment of the inflow cannula during implantation. The difficulty remains in predicting the final position of the inflow cannula post-implant. There are several reasons that influence the final position of the inflow cannula, such as the pressure of the thoracic wall, size of the left ventricle, gravity, late remodelling of the left ventricle and the angle of the inflow cannula [5]. Ideally, a standardized method is developed to optimize the position of the inflow cannula pre-implantation. Our study observed that septal–lateral angulation was most notably affecting the occurrence of low-flow alarms. Based on this result, it could be recommended to place the inflow cannula parallel to the septal wall, directed towards the coaptation of the mitral valve, thereby reducing suboptimal suction of the LVAD. This recommendation is supported by intraventricular flow dynamics during LVAD support investigated in earlier studies [14, 16, 17]. Current technologies such as reconstructed intraventricular flow dynamics and planning the procedure with virtual reality, a hybrid room or three-dimensional printed models could provide the pre-implantation information needed for the optimal alignment of the inflow cannula.

### Standardized measuring method of the inflow cannula

To evaluate the angle of the inflow cannula, a standardized method is essential to investigate this association. A few methods are described in the literature to measure and calculate this angle [12, 18, 19]. However, these methods differ widely and a standardized measurement technique has not clearly been described. Our study describes very clearly a measurement protocol that is practical. The inflow cannula is a three-dimensional object and should thereby be evaluated in multiplanar views. A chest X-ray would therefore be insufficient and not adequate to show to structures of the heart. To evaluate the proposed ‘optimal’ flow line from the coaptation of the mitral valve and the apex, the mitral valves need to be displayed, which is in concordance with other studies. This requires a contrast-enhanced chest CT scan.

### Clinical implications

This study shows clearly the haemodynamic sequelae of the inflow cannula angulation on adequate LVAD flow and subsequent risk of relapsing low-flow alarms. This emphasizes the importance of optimal intraoperative alignment of the inflow cannula to reduce the possibility of relapsing low-flow alarms during support. Furthermore, the angulation of the inflow cannula could be influenced by potential chest wall abnormalities or the effect of the sternum closure at the end of the implantation. If a patient is suffering from relapsing low-flow alarms during follow-up, a contrast-enhanced CT scan should be the primary diagnostic tool to review the influence of the inflow cannula angulation. At our centre, surgeons are now aware of the optimal alignment of the inflow cannula and a contrast-enhanced CT scan is performed at patients when suffering from relapsing low-flow alarms.

### Limitations

Some limitations should be taken into consideration. First, when a patient is suffering from relapsing low-flow alarms they do not always contact the hospital with possible detection bias. However, the data log is read out every visit and is very likely that most of the low-flow alarms are captured. Second, our study included all CT scan regardless of the reason. However, the number of patients was limited and, therefore, we aimed to include as many patients and CT scans as possible. Serial scans were limited in the study population and, therefore, we have not evaluated the influence of loading conditions, pharmacological agents or left ventricular remodelling on inflow cannula position. Furthermore, the ROC analysis could not account for relapsing low-flow alarms over time, and thereby only the event of having low-flow alarms could be evaluated in the analysis for the optimal cut-off point, resulting in a low sensitivity and specificity. Finally, due to the retrospective nature and limited sample size, the influence of low body mass index or body surface area on the inflow cannula angulation remains to be elucidated in future studies. Still, the study is original and the hypothesis is biologically plausible.

## CONCLUSION

This study shows an increase in the number of low-flow alarms over time when the degrees of the angular position of the LVAD inflow cannula expand, specifically in the septal–lateral plane. This emphasizes the importance of the LVAD inflow cannula angular position to prevent relapsing low-flow alarms and risk of diminished quality of life and increased morbidity. The influence of both septal–lateral angulation and relapsing low-flow alarms on postoperative adverse events remains to be elucidated with larger series.

## SUPPLEMENTARY MATERIAL

Supplementary material is available at *EJCTS* online.

## Funding

The authors received no funding for this work.

**Conflict of interest:** none declared.

## Supplementary Material

ezac415_Supplementary_Data

## Data Availability

All relevant data are available on request from the authors.

## ABBREVIATIONS

CT: Computed tomography
HF: Heart failure
IQR: Interquartile range
LVAD: Left ventricular assist device
ROC: Receiver operating characteristic
