# Supplementary material for: Relapsing low-flow alarms due to suboptimal alignment of the left ventricular assist device inflow cannula
Source: Eur J Cardiothorac Surg. 2022 Aug 22;62(4):ezac415. doi: 10.1093/ejcts/ezac415 (PMC9789739; doi:10.1093/ejcts/ezac415)
Supplement: ezac415_Supplementary_Data [file ezac415_supplementary_data.docx]

**SUPPLEMENTARY MATERIAL**

**Table 1.** Angular position of the inflow cannula divided according to the cardiac phase diastolic versus systolic. Presented as median with Interquartile Range (IQR).

|  | **Diastolic (n = 15)** | **Systolic (n = 33)** | ***p*** |
| --- | --- | --- | --- |
| **Septal-lateral** | 20.0 [15.5, 29.8] | 24.0 [7.5, 30.0] | 0.850 |
| **Anterior-posterior** | 25.0 [11.50, 33.00] | 17.0 [5.0, 30.0] | 0.504 |
| **Maximal plane** | 32.5 [27.0, 38.0] | 31.0 [23.5, 38.0] | 0.609 |

**Table 2.** Clinical outcomes presented with Hazard Ratio (HR) and 95% Confidence Intervals (CI) according to the three different planes, septal-lateral, anterior-posterior, and maximal angle of the inflow cannula in any direction, divided by severity of the angulation according to the optimal cut-off point.

|  | **Septal-lateral**  **<28 and >28 degrees** | | **Anterior-posterior**  **<13.5 and >13.5 degrees** | | **Maximal inflow cannula angulation**  **<31.5 and >31.5 degrees** | |
| --- | --- | --- | --- | --- | --- | --- |
|  | **HR (CI)** | ***P*** | **HR (CI)** | ***P*** | **HR (CI)** | ***P*** |
| **Readmission** | 1.65 (0.80 – 3.42) | 0.176 | 1.04 (0.51 – 2.10) | 0.924 | 1.57 (0.76 – 3.21) | 0.221 |
| **Right heart failure** | 0.57 (0.06 – 5.09) | 0.612 | 0.96 (0.16 – 5.76) | 0.965 | 0.74 (0.12 – 4.47) | 0.747 |
| **Bleeding** | 1.17 (0.66 – 15.69) | 0.149 | 0.87 (0.19 – 3.94) | 0.854 | 3.26 (0.63 – 16.93) | 0.159 |
| **Neurological dysfunction** | 1.88 (0.17 – 20.91) | 0.607 | - | - | 0.53 (0.05 – 5.86) | 0.605 |
| **Death** | 2.64 (0.71 – 9.86) | 0.149 | 0.78 (0.21 – 2.94) | 0.716 | 1.23 (0.33 – 4.59) | 0.755 |


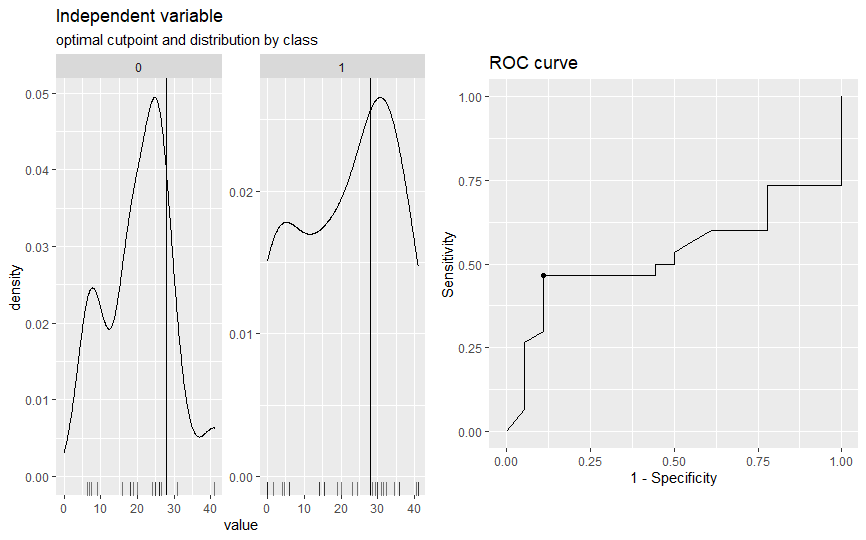


**Figure 1.** ROC analysis of septal-lateral angulation with optimal cut-off point calculation between group low flow alarm(s) (1) or no low flow alarm (0). Optimal cut-off point is calculated at 28 degrees. N = 18 in < 28 degrees group, n = 30 in > 28 degrees group. Area under the ROC curve (AUC) = 0.5269.


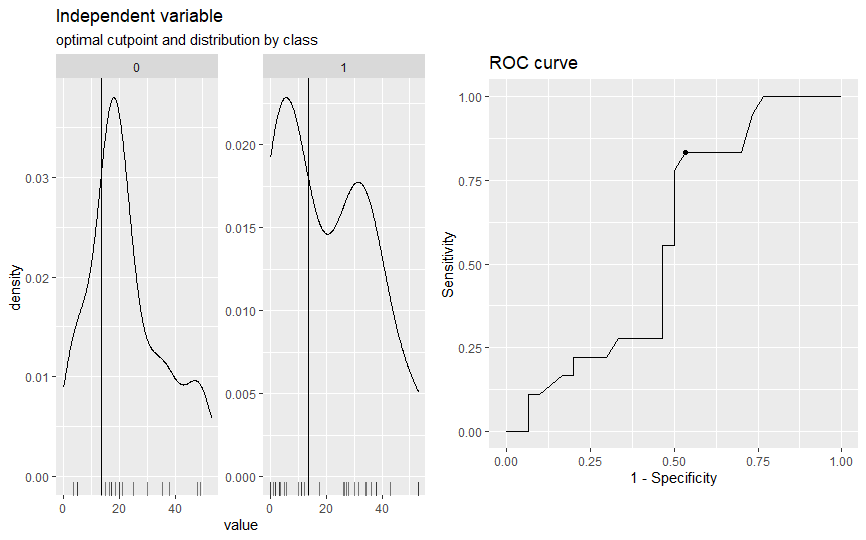


**Figure 2.** ROC analysis of combined anterior-posterior angulation with optimal cut-off point calculation between group low flow alarm(s) (1) or no low flow alarm (0). Optimal cut-off point is calculated at 13.5 degrees. N = 30 in < 13.5 degrees group, n = 18 in > 13.5 degrees group. Area under the ROC curve (AUC) = 0.5657.

**
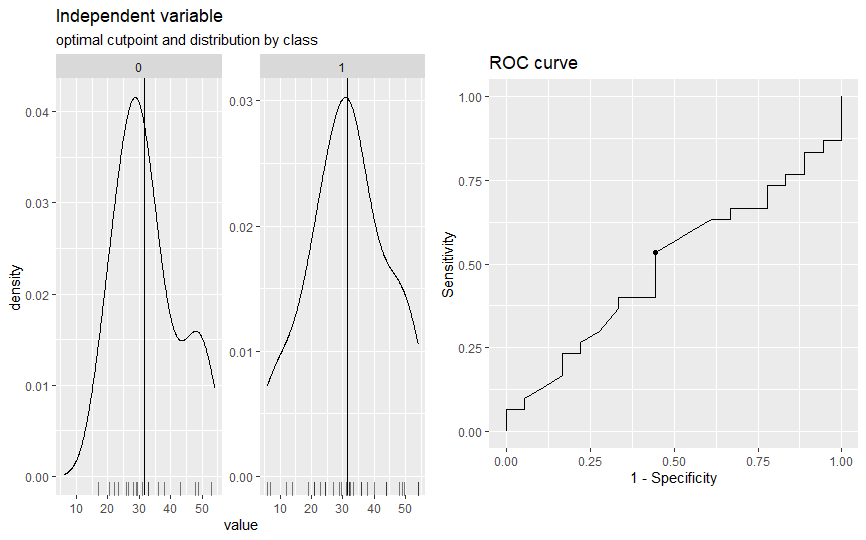
**

**Figure 3.** ROC analysis of maximal angulation of the inflow cannula with optimal cut-off point calculation between group low flow alarm(s) (1) or no low flow alarm (0). Optimal cut-off point is calculated at 31.5 degrees. N = 18 in < 31.5 degrees group, n = 30 in > 31.5 degrees group. Area under the ROC curve (AUC) = 0.4944.
